# Teenage Pregnancy and Its Adverse Obstetric and Perinatal Outcomes at Lemlem Karl Hospital, Tigray, Ethiopia, 2018

**DOI:** 10.1155/2020/3124847

**Published:** 2020-01-19

**Authors:** Ayele Mamo Abebe, Girma Wogie Fitie, Desalegn Abebaw Jember, Mihretab Mehari Reda, Getu Engida Wake

**Affiliations:** ^1^Department of Nursing, Debre Berhan University, Debre Birhan, Amhara, Ethiopia; ^2^Department of Midwifery, Wollo University, Dessie, Amhara, Ethiopia; ^3^Department of Midwifery, Health Science College, Mekelle University, Mekelle City, Tigray, Ethiopia; ^4^Department of Midwifery, Health Science College, Debre Birhan University, Debre Birhan, Amhara, Ethiopia

## Abstract

**Introduction:**

One of the major public health issues across the whole world these days is teenage pregnancy which is defined as being pregnant in the age ranging from 13–19 years. About 11% of all births which occurred worldwide accounted for girls aged 15 to 19 years. From these, 95% teenage deliveries occur in low- and middle-income countries. World health 2014 statistics show that complications during pregnancy and childbirth are the second cause of death for 15–19-year-old girls globally. The aim of this study was to determine adverse obstetrical and perinatal outcomes of teenage pregnancy among deliveries at Lemlem Karl General Hospital, Tigray, Ethiopia, 2018.

**Result:**

This study result showed that 17.5% of the teenagers and 6.8% of the adults deliver low birth weight neonates. From the total teenage mothers, about thirty-five (11.3%) of them had developed pregnancy-induced hypertension, whereas about thirteen (4.2%) of adults develop pregnancy-induced hypertension. Regarding cesarean delivery, forty (12.9%) of those teenage mothers deliver by a cesarean section while 58 (18%) of the adult mothers deliver by cesarean delivery. Teenage pregnancy was significantly associated with adverse obstetric and perinatal outcomes, a cesarean delivery (AOR: 0.57; 95% CI, 0.36–0.90), episiotomy (AOR: 2.01; 95% CI, 1.25–3.39), and low birth weight (AOR: 2.22; 95% CI, 1.13–4.36), and premature delivery were 2.87 (1.49–5.52). This study shows that adverse obstetric and perinatal outcomes were significantly associated with teenagers than adult mothers. Therefore, giving health education on focused ANC is very important to bring change to the teenager at this study area.

## 1. Introduction

One of the major public health issues across the whole world is teenage pregnancy which is defined as pregnancy in girls aged 13–19 years [1, 2]. According to WHO, seven countries mainly constitute for half of all adolescent births, namely, Bangladesh, Brazil, the Democratic Republic of the Congo, Ethiopia, India, Nigeria, and the United States of America [1, 3, 4].

Determinants of teenage pregnancy in a study employed in developing countries included the following reasons as attributing factors for teenage pregnancy; lack of knowledge on sexuality education, ineffective utilization of modern contraceptives, cultural obedience, socioeconomic dependence of females on males, and peer influence [5–7].

Teenage pregnancy is greatly associated with adverse pregnancy outcomes like obstructed labor, pre-eclampsia, anemia, operative deliveries, puerperal endometritis, postpartum hemorrhage, low birth weight, preterm delivery, and perinatal death [8–15].

Studies conducted in North India and Turkey outlined that babies of adolescent mothers were 1.65 and 4.94 times more likely to be born prematurely and face intrauterine fetal death (IUFD), respectively [16, 17].

Studies conducted in Taiwan, USA, and Korea showed that teenagers were 1.58, 1.36, and 1.16 times more likely to have preterm delivery than the adults, respectively [18–20]. A study in Nepal showed that low birth weight in teenagers and adults was 24% vs. 9% (*P*=0.013) [21]. Another study was conducted in Ankara in a referral hospital revealed that teenagers deliver 4.14 times more likely prematurely than adults [22].

A study conducted in Cameron revealed that subjects with teenage pregnancy were 1.94 and 1.46 times more likely to deliver very low birth weight and low birth weight neonates than adults [23]. Studies showed that a significantly higher rate of cesarean delivery was found among adult mothers compared with teenage mothers [24, 25]. In a study conducted in Sweden, teenagers were 30% less likely to develop postpartum hemorrhage (PPH) and 63% less probable to be complicated by Antepartum hemorrhage (APH) secondary to placenta Previa [26]. As a study in Finland showed, teenagers are 1.2 and 3.2 times more likely to develop preeclampsia and eclampsia than adults [27]. Even though a lot has been said about teenage pregnancy and its associated factors and some deals about the outcomes across the globe, as far as the investigator's knowledge, there are limited published researches on adverse obstetric and perinatal outcomes of teenage pregnancy in Ethiopia. In addition, there are three studies on teenage pregnancy and associated factors in Ethiopia. But these studies had not touched about the negative outcome of the teenage pregnancy. Therefore, the aim of this study was to assess teenage pregnancy and its adverse obstetric and perinatal outcomes at Lemlem Karl hospital, Tigray, Ethiopia, 2018.

## 2. Methods and Materials

### 2.1. Study Area and Study Period

This retrospective cohort study design was conducted at Lemlem Karl general hospital which is found at Maychew town, southern zone of Tigray regional state, northern Ethiopia, at a distance of 662 km from Addis Ababa to the North. Maychew town has a total population of 36,925 and has four kebeles which are located in the southern part of Tigray but in the northern part of Ethiopia [28]. The study was conducted from November 2017 to December 2017.

### 2.2. Sample Size

The sample size was calculated using the double population proportion formula considering an assumption of 95% confidence level, power 80%, the proportion of low birth weight in teenage pregnancy is 9.5%, and adult pregnancy is 3.6% taken from a study conducted in Cameron [29], and then using Epi info statistical software, the final sample size calculated gives 618 (309 teenagers and 309 adults) with 1 : 1 ratio. Six hundred and eighteen is the highest sample size that the principal investigator calculated for different outcome variables by using the Epi info statistical software (Table 1).

### 2.3. Sampling Technique

There were a total of 4,683 deliveries from September 2014 to September 2017 GC. Of these, 932 and 3,089 were teenagers and adult deliveries, respectively. Records were reviewed from the delivery registration book of the obstetrics ward and registration logbook used as a sampling frame. Then, the investigator would have two different *K* values for teenagers and for adults. Calculating the *K* (*N*/*n* = 932/309 ≈ 3), value for teenagers where “*N*” is total delivered teenage mothers admitted in the ward during the three years period and “*n*” is calculated sample size for teenagers, then study cards were selected in every 3rd interval from the registration book of teenager's in the three years record. Similarly, the investigator calculate the *K* value for the adults, the *K* (*N*/*n* = 3089/309 ≈ 10), value for adults where “*N*” is total delivered adult mothers admitted in the ward during the three years period, and “*n*” is calculated sample size for adults, and then study cards were selected in every 10th interval from the registration book of adults in the three years period. The first study card/chart was selected randomly for both teenagers and adults from their own frame.

### 2.4. Data Collection Tools and Procedures

Data were collected from the patients/clients card by reviewing retrospectively from the year 2014–2017 GC. One master in clinical midwifery student, two BSc midwifery professionals, and two diploma midwifery professionals were oriented by the principal investigator about the purpose of the study and how to collect the data, as well as how to fill the questionnaire properly.

### 2.5. Data Quality Assurance

A checklist was prepared in English language by the principal investigator and reviewed by the advisors. The checklist was pretested in 31 (5%) of the mothers' charts at Lemlem Karl general hospital before going to the actual data collection. The collected data was checked by the supervisor and principal investigator daily for completeness.

### 2.6. Data Analysis Procedure

The data were coded, entered, cleaned with Epi info version 3.5.1, and analyzed by SPSS version 20. In multivariate logistic regression analysis, variables having a *P* value less than or equal to 0.05 were considered to have a statistically significant association with the dependent variable, and the result was presented with different tables and graph.

## 3. Results

### 3.1. Sociodemographic Information of Teenage and Adult Mothers

A total of 618 charts of women who delivered at Lemlem Karl general hospital were reviewed, who met the eligibility criteria of this study. Of these, 309 were charts of teenage while the rest were adult women charts. The median age of the study population was 21, the 75th percentile was 27 years, and 25th was 19. So, IQR = 27–19, 50% of the study populations were found in the age range of 19–27 years. The majority of them from both age groups were unemployed which was about 89% and 71.8% for teenagers and adults, respectively.

Regarding the obstetric characteristics, more than three-quarters of teenagers 260 (84.1%) and about 80 (25.9%) of adults were primigravida. Concerning to antenatal care visits, more than three-quarters of the study populations had at least one antenatal care (ANC) visits during their current pregnancy time, and teenagers had lower ANC follow-up than adults, 77.3% and 83.2%, respectively (Table 2).

### 3.2. Obstetric Outcomes of Teenage and Adult Mothers at Lemlem Karl General Hospital

From the total teenage mothers, about thirty-five (11.3%) of them had to develop pregnancy-induced hypertension, whereas about thirteen (4.2%) of adults developed pregnancy-induced hypertension.

Episiotomy was performed for more than one-quarter of the teenager's delivery and about twelve percent of adult delivery. In relation to the gestational age at delivery, 14 (4.5%) and 29 (9.4%) of the teenage and adult had post-term delivery, respectively (Table 3).

### 3.3. Perinatal Outcomes of Teenage and Adult Mothers at Lemlem Karl General Hospital

Concerning to adverse perinatal outcomes of teenage delivery, about 12.9% delivered prematurely whereas in adult-only, about 4.5% delivered prematurely. Regarding birth weight of the newborn, teenagers deliver low birth weight neonates more than double of the adult deliveries (Table 4).

### 3.4. Association of Adverse Obstetric Outcomes with Maternal Age

Teenagers developed pregnancy-induced hypertension 2.29 times more likely than the adult mothers (AOR: 2.29; 95% CI, 1.01–5.19). Teenagers were 43% less likely to undergo cesarean delivery than the adult mothers (AOR: 0.57; 95% CI, 0.36–0.90). Teenagers were 2.01 times more likely to have episiotomy than the adult mothers (AOR: 2.01; 95% CI, 1.25–3.39). The probability of teenagers to deliver post-term was 31.5% (OR: 0.46; 95% CI, 0.24–0.89) (Table 5).

### 3.5. Association of Adverse Perinatal Outcomes with Maternal Age

Adverse perinatal outcomes revealed that teenage mothers were 2.87 times more likely to deliver prematurely than the adult mothers (AOR: 2.87; 95% CI, 1.49–5.52). Chance of giving low birth weight was more than double in teenagers as compared with the adult age group mothers (AOR: 2.22; 95% CI, 1.13–4.36). A severe neonatal condition was also more pronounced in teenage groups than the adults which was about 2.98 times more likely (AOR: 2.98; 95% CI, 1.25–7.14) (Table 6).

## 4. Discussion

According to this study, 17.5% of the teenagers deliver low birth weight and 6.8% of the adults deliver low birth weight neonates. Teenagers were 2.22 times more likely to deliver low birth weight neonates than adult mothers (AOR: 2.22; 95% CI, 1.13–4.36). This study was aligned with a study conducted in North India, in Cameron, and in Egypt [16, 23, 29]. This might be due to the anatomical immaturity, and continued growth of the teenagers may represent the biological growth barrier to their fetus and the low socioeconomic status.

According to this study, the result shows that teenagers were 2.87 times more likely to deliver prematurely than adult mothers (AOR: 2.87; 95% CI, 1.49–5.52). The same finding was observed in studies conducted in India, Turkey, Taiwan, Korea, and Cameron [10, 16–18, 23, 29]. The possible reason for this might be that teenagers were highly exposed to psychological instability/stress because of cultural, social, and economic factors in their living environment. Despite our finding, studies conducted in Sweden (AOR: 1.03; 95% CI, 0.98–1.09) and South Africa (*P*=0.702) showed there was no significant difference in preterm deliveries between the two age groups [24, 31]. This could be attributed by the study area setting and difference in sample sizes.

Based on this study, teenagers were 2.98 times more likely to have severe neonatal conditions than adult mothers (AOR: 2.98; 95% CI, 1.25–7.14). This result was similar with an institutional-based cross-sectional study conducted by the average health organization [10]. The possible explanation might be a socioeconomic condition, health service utilization, and nutritional status of mothers.

In this study, teenagers develop pregnancy-induced hypertension 2.29 times more likely than adult mothers (AOR: 2.29; 95% CI, 1.01–5.19). Our finding was supported the study conducted in the USA [19]. This might be attributed to the fact that null parity and age less than 20 years are the possible risk factors for the development of pregnancy-induced hypertension. However, our finding was inconsistent with studies conducted in Pakistan and Ankara [22, 25]. This difference might be explained by the difference in health-care service utilization and preconception care especially folic acid supplementation.

Regarding cesarean delivery, teenagers were 43% less likely to deliver by cesarean delivery than adult mothers (AOR: 0.57; 95% CI, 0.36–0.90). This study had a similar finding with studies conducted in Sweden and USA [19, 31]. Another study conducted in Ankara, Turkey also showed that teenagers were 36% less likely to undergo cesarean delivery than adults (AOR: 0.64; 95% CI, 0.64–0.89) [22]. The possible explanation for this might be due to the awareness difference between the two age groups about cesarean delivery.

However, this study finding has contradiction with three studies conducted in Cameron which were conducted at different time periods [6, 23, 29]. This variation might be due to the difference in sample sizes and study area setting. In this study, episiotomy was performed about two times more likely on teenagers than adults. Our finding is similar to the studies conducted in Cameron, Turkey, Romania, and Cameron [6, 19, 27, 32]. This might be due to the study area setting and the rules to apply episiotomy.

## 5. Conclusion

This study shows that adverse obstetric and perinatal outcomes were significantly associated with teenage mothers than adult mothers like pregnancy-induced hypertension and episiotomy. Low birth weight, preterm delivery, and severe neonatal conditions are also clearly more significant in teenagers.

## Figures and Tables

**Table 1:** Sample size calculated by Epi info statistical software from different outcome variables based on their proportion for teenagers and adult mothers (in 1 : 1 ratio of teenage and adult), 2018 (*n*=618).

Variable	Proportion of outcomes in exposed	Proportion of outcomes in nonexposed	OR	Sample	Reference
Cesarean section	18.5	9.5	1.99	Exposed 255	Ikeako et al. [30]
				Nonexposed 255	
				Total **510**	

Low birth weight	9.5	3.6	2.81	Exposed 309	Egbe et al. [29]
				Nonexposed 309	
				Total **618**	

Preterm	20	7	3.32	Exposed 123	Tripathi and Sherchand [21]
				Nonexposed 123	
				Total **246**	

**Table 2:** Sociodemographic information of teenage and adult mothers in Lemlem Karl general hospital, Tigray region, Ethiopia, 2018 (*n*=618).

Variable	Response	Age category		Total *N* (%)
		Teenage *N* (%)	Adults *N* (%)	
Residence	Urban	103 (33.3)	196 (63.4)	299 (48.4)
	Rural	206 (66.7)	113 (36.6)	319 (51.6)

Employment	Unemployed	275 (89.0)	222 (71.8)	497 (80.4)
	Employed	34 (11.0)	87 (28.2)	121 (19.6)

Marital status	Married	278 (90.0)	297 (96.1)	575 (93.0)
	Single	31 (10.0)	12 (3.9)	43 (7.0)

Gravidity	Multigravida	49 (15.9)	229 (74.1)	278 (45.0)
	Primigravida	260 (84.1)	80 (25.9)	340 (55.0)

ANC	Yes	239 (77.3)	257 (83.2)	496 (80.3)
	No	70 (22.7)	529 (16.8)	122 (19.7)

**Table 3:** Obstetric outcomes of teenage and adult mothers delivered at Lemlem Karl general hospital, Tigray region, Ethiopia, 2018 (*n*=618).

Variable	Response	Age category		Total *N* (%)
		Teenage *N* (%)	Adults *N* (%)	
PIH	No	274 (88.7)	296 (95.8)	570 (92.2)
	Yes	35 (11.3)	13 (4.2)	48 (7.8)

Cesarean delivery	No	269 (87.1)	251 (81.2)	520 (84.1)
	Yes	40 (12.9)	58 (18.8)	98 (15.9)

Instrumental delivery	No	283 (91.6)	282 (91.2)	565 (91.4)
	Yes	26 (8.4)	27 (8.8)	53 (8.6)

PPH	No	297 (96.2)	292 (94.5)	593 (96.0)
	Yes	12 (3.8)	17 (5.5)	25 (4.0)

APH	No	298 (96.4)	291 (94.2)	589 (95.3)
	Yes	11 (3.6)	18 (5.8)	29 (4.7)

Perineal tear	No	286 (92.6)	293 (94.8)	579 (93.7)
	Yes	23 (7.4)	16 (5.2)	39 (6.3)

Post-term delivery	No	295 (95.5)	280 (90.6)	575 (93.0)
	Yes	14 (4.5)	29 (9.4)	43 (7.0)

Episiotomy	No	214 (69.3)	272 (88.0)	486 (78.6)
	Yes	95 (30.7)	37 (12.0)	132 (21.4)

**Table 4:** Perinatal outcomes of teenage and adult mothers delivered at Lemlem Karl general hospital, Tigray region, Ethiopia, 2018 (*n*=618).

Variable	Response	Age category		Total *N* (%)
		Teenage *N* (%)	Adults *N* (%)	
Preterm delivery	No	269 (87.1)	295 (95.5)	564 (91.3)
	Yes	40 (12.9)	14 (4.5)	54 (8.7)

Low birth weight	No	255 (82.5)	288 (93.2)	543 (87.9)
	Yes	54 (17.5)	21 (6.8)	74 (12.1)

Still birth/IUFD	No	290 (93.9)	299 (96.8)	589 (95.3)
	Yes	19 (6.1)	10 (3.2)	29 (4.7)

SNC	No	276 (89.3)	298 (96.4)	574 (92.9)
	Yes	33 (10.7)	11 (3.6)	44 (7.1)

Fetal distress	No	294 (95.1)	296 (95.8)	590 (95.5)
	Yes	15 (4.9)	13 (4.2)	28 (4.5)

**Table 5:** Bivariate and multivariate analysis of the adverse obstetrical outcomes of teenagers and adult mothers delivered at Lemlem Karl general hospital, Tigray region, Ethiopia, 2018 (*n*=618).

Variable	Category	Obstetric outcomes PIH		COR (95% CI)	AOR (95% CI)	*P* value
		Yes *N* (%)	No *N* (%)			
Age	Teenage	35 (72.9)	274 (48.1)	2.91 (1.51–5.61)	2.29 (1.01–5.19)	0.048
	Adult	13 (27.1)	296 (51.9)	1	1	
Residence	Rural	32 (66.7)	287 (50.4)	1.97 (1.06–3.67)	1.51 (0.79–2.88)	0.217
	Urban	16 (33.3)	283 (49.6)	1	1	
Gravidity	Primigravida	34 (70.8)	306 (53.7)	2.1 (1.10–3.99)	1.18 (0.54–2.59)	0.69
	Multigravida	14 (29.2)	264 (46.3)	1	1	
ANC	No	15 (31.2)	107 (18.8)	0.51 (0.27–0.97)	0.56 (0.29–1.07)	0.079
	Yes	33 (68.8)	463 (81.2)	1	1	

*Cesarean delivery*						
Age	Teenage	40 (40.8)	269 (51.7)	0.64 (0.415–0.997)	0.57 (0.36–0.90)	0.016^*∗*^
	Adult	58 (59.2)	251 (48.3)	1	1	
Residence	Rural	58 (59.2)	261 (50.2)	1.5 (0.970–2.319)	1.78 (1.12–2.82)	0.014
	Urban	40 (40.8)	259 (49.8)	1	1	

*Instrumental delivery*						
Age	Teenage	26 (49.1)	283 (50.1)	0.96 (0.55–1.69)	—	0.886
	Adult	27 (50.9)	282 (49.9)	1		
Gravidity	Primigravida	41 (78.8)	299 (52.8)	3.040 (1.564–5.905)	3.004 (1.544–5.843)	0.001
	Multigravida	11 (21.2)	267 (47.2)	1	1	

*Episiotomy*						
Age	Teenage	95 (72)	214 (44)	3.26 (1.39–3.33)	2.01 (1.25–3.39)	0.004^*∗*^
	Adult	37 (28)	272 (56)	1	1	
Gravidity	Primigravida	85 (79.4)	255 (49.9)	2.88 (2.35–6.39)	2.35 (1.39–3.96)	0.001
	Multigravida	22 (20.6)	256 (50)	1	1	

*Postpartum hemorrhage*						
Age	Teenage	12 (41.4)	297 (50.4)	0.69 (0.33–1.48)	—	0.96
	Adult	17 (68)	292 (49.2)	1		

*APH*						
Age	Teenage	11 (37.9)	298 (50.6)	0.597 (0.28–1.29)	0.82 (0.31–2.2)	0.698
	Adult	18 (62.1)	291 (49.4)	1		
Residence	Rural	21 (72.4)	298 (50.6)	2.56 (1.12–5.9)	3.1 (1.32–7.44)	0.009
	Urban	8 (27.6)	291 (49.4)	1	1	
Gravidity	Multigravida	20 (69)	258 (43.8)	1	1	
	Primigravida	9 (31%)	331 (56.2)	0.35 (0.16–0.78)	0.34 (0.125–0.918)	0.033

*Perennial tear*						
Age	Teenage	23 (59)	286 (49.4)	1.47 (0.762.85)	—	0.249
	Adult	16 (41)	293 (50.6)	1		

*Post-term delivery*						
Age	Teenage	14 (32.6)	295 (51.3)	0.46 (0.24–0.89)		0.02
	Adult	29 (67.4)	280 (48.7)	1		

**Table 6:** Bivariate and multivariate analysis of the adverse perinatal outcomes of teenagers and adult mothers delivered at Lemlem Karl general hospital, Tigray region, Ethiopia, 2018 (*n*=618).

Variable	Category	Perinatal outcomes of preterm delivery		COR (95% CI)	AOR (95% CI)	*P* value
		Yes *N* (%)	No *N* (%)			
Age	Teenage	40 (74.1)	269 (47.7)	3.13 (1.67–5.89)	2.87 (1.49–5.52)	0.002^*∗*^
	Adult	14 (25.9)	295 (51.3)	1	1	
Residence	Rural	35 (64.8)	284 (50.4)	1.8 (1.04–3.25)	1.35 (0.74–2.49	0.33
	Urban	19 (35.2)	280 (49.6)	1	1	
APH	Yes	7 (13)	22 (3.9)	3.67 (1.49–9.03)	4.9 (1.9–12.9)	0.001
	No	47 (87)	542 (95.1)	1	1	
PIH	Yes	11 (20.4)	37 (6.6)	3.64 (1.74–7.65)	3.02 (1.39–6.6)	0.005
	No	43 (79.6)	527 (93.4)	1	1	

*Low birth weight*						
Age	Teenage	54 (72)	255 (47)	2.91 (1.71–4.94)	2.22 (1.13–4.36)	0.021^*∗*^
	Adult	21 (28)	288 (53)	1	1	
Residence	Rural	54 (73)	265 (48.7)	2.84 (1.66–4.88)	2.27 (1.29–3.97)	0.004
	Urban	20 (27)	279 (51.3)			
Gravidity	Primigravida	50 (67.6)	29 (53.3)	1.83 (1.09–3.05)	1.04 (0.55–1.97)	0.911
	Multigravida	24 (32.4)	254 (46.7)	1	1	
PIH	Yes	19 (25.3)	29 (5.3)	6.1 (3.23–11.66)	4.89 (2.53–9.52)	0.001
	No	56 (74.7)	514 (94.7)	1	1	

*Still birth/IUFD*						
Age	Teenage	19 (65.5)	290 (49.2)	1.96 (0.89–4.28)	—	0.43
	Adult	10 (34.5)	299 (50.8)	1		

*SNC*						
Age	Teenage	33 (75.0)	276 (48.1)	3.24 (1.606–6.534)	2.98 (1.25–7.14)	0.014^*∗*^
	Adult	11 (25.0)	298 (51.9)	1	1	
Residence	Rural	28 (70)	291 (50.3)	2 (1.10–4.10)	1.58 (0.797–3.12)	0.19
	Urban	12 (30)	287 (49.7)	1	1	
Gravidity	Primigravida	30 (75)	310 (53.6)	1.83 (0.95–3.51)	0.92 (0.41–2.05)	0.837
	Multigravida	10 (25)	268 (46.4)	1	1	
PIH	Yes	41 (7)	7 (15.9)	2.46 (1.03–5.86)	1.59 (0.63–4.00)	0.326
	No	533 (93)	37 (84.1	1	1	

*Fetal distress*						
Age	Teenage	15 (53.6)	294 (49.8)	1.16 (0.54–2.48)	—	0.699
	Adult	13 (46.4)	296 (50.2)	1		

## Data Availability

Data supporting the conclusions of this article are available by request to Ayele Mamo. The questionnaire and full research will be made available to researchers wishing to use them for noncommercial purposes. The Supplementary data will be put below the acknowledgement.

## Abbreviations

APH: Antepartum hemorrhage
ANC: Antenatal care
AOR: Adjusted odds ratio
CPD: Cephalopelvic disproportion
EDHS: Ethiopian demographic health survey
ETB: Ethiopian birr
GA: Gestational age
GC: Gregorian calendar
GP: General practitioner
IESO: Integrated, emergency surgery and obstetrics
IUFD: Intrauterine fetal death
OB/GYN: Obstetrics/gynecology
PPH: Postpartum hemorrhage
SNC: Severe neonatal condition
SPSS: Statistical package for social science
SVD: Spontaneous vertex delivery
UN: United Nation
WHO: World Health Organization.

## Additional Points

*Limitations*. Factors like economic status, educational level, and other unmentioned factors which might affect the outcomes of teenage and adult pregnancy outcomes could not be addressed in this study.
